# Low Dose Focused Ultrasound Induces Enhanced Tumor Accumulation of Natural Killer Cells

**DOI:** 10.1371/journal.pone.0142767

**Published:** 2015-11-10

**Authors:** Naomi S. Sta Maria, Samuel R. Barnes, Michael R. Weist, David Colcher, Andrew A. Raubitschek, Russell E. Jacobs

**Affiliations:** 1 Division of Biology and Biological Engineering, Beckman Institute, California Institute of Technology, Pasadena, CA, United States of America; 2 Department of Cancer Immunotherapeutics and Tumor Immunology, Beckman Institute, City of Hope, Duarte, CA, United States of America; University of Pavia, ITALY

## Abstract

Natural killer (NK) cells play a vital antitumor role as part of the innate immune system. Efficacy of adoptive transfer of NK cells depends on their ability to recognize and target tumors. We investigated whether low dose focused ultrasound with microbubbles (*ldb*FUS) could facilitate the targeting and accumulation of NK cells in a mouse xenograft of human colorectal adenocarcinoma (carcinoembryonic antigen (CEA)-expressing LS-174T implanted in NOD.Cg-Prkdc^scid^Il2rg^tm1Wjl^/SzJ (NSG) mice) in the presence of an anti-CEA immunocytokine (ICK), hT84.66/M5A-IL-2 (M5A-IL-2). Human NK cells were labeled with an FDA-approved ultra-small superparamagnetic iron oxide particle, ferumoxytol. Simultaneous with the intravenous injection of microbubbles, focused ultrasound was applied to the tumor. *In vivo* longitudinal magnetic resonance imaging (MRI) identified enhanced accumulation of NK cells in the ensonified tumor, which was validated by endpoint histology. Significant accumulation of NK cells was observed up to 24 hrs at the tumor site when ensonified with 0.50 MPa peak acoustic pressure *ldb*FUS, whereas tumors treated with at 0.25 MPa showed no detectable NK cell accumulation. These clinically translatable results show that *ldb*FUS of the tumor mass can potentiate tumor homing of NK cells that can be evaluated non-invasively using MRI.

## Introduction

Natural killers (NK) cells are critical components of the immune system that show promise in cancer immunotherapy based on their ability to lyse malignant and infected cells without prior sensitization or immunization [1, 2]. NK cells have the ability to discriminate between “normal self” and “altered-self” through MHC class I—specific inhibitory receptors and activating receptors (“missing-self recognition”) [1]. It is the balance between inhibitory and activating receptors that direct NK cell activity. Activating receptors recognize upregulated proteins or pathogen-encoded ligands expressed by infected or tumor transformed cells and not by the host cells [1, 3].

NK cells are categorized into two subsets depending on the level of CD56 expression [4]. CD56^dim^ NK cells are predominantly cytolytic and often express high levels of low-affinity Fc receptor for IgG (FcγIIIR–CD16) that allows them to recognize antibodies on target cells and trigger NK cell mediated antibody-dependent cell-mediated cytotoxicity (ADCC). Monoclonal antibody treatments of non-Hodgkin lymphoma (with anti-CD20 –Rituximab) and metastatic breast cancer (with anti-trastuzumab/Herceptin) have shown that ADCC is NK cell-mediated [5–7]. CD56^bright^ NK cells have mainly modulatory roles by producing cytokines that activate cells of the immune system through their cytokine receptors. Cytotoxic and cytokine production functions of NK cells can impact downstream members of both the innate and adaptive immune systems, such as dendritic cells, macrophages, neutrophils, and T and B cells, and promote suppression of infected and tumor cells. Further, interaction with other immune cells, such as dendritic cells, and effector mechanisms like interferons and cytokines (IL-1, IL-12, IL-15, IL-18, and IL-21) can also potentiate NK cell activity, surveillance, development and maturation [1, 3].

The use of NK cells in immunotherapy [8] is being explored through alteration of NK cells for adoptive transfer [9] as well as modification of the immune and tumor environment using antibodies, cytokines, chemokines, and physical manipulation via focused ultrasound [9, 10]. *Ex vivo* activated and expanded autologous NK cells that are adoptively transferred back to patients have shown some success in NK cells tumor accumulation and tumor cell killing [11]. Treatments with allogenic NK cells have also shown greater NK cell activity through killer cell like inhibitory receptor (KIR) mismatch, which contributes to the alloreactivity of NK cells via the “missing-self recognition” mechanism [12, 13]. Alternatively, NK cell lines, which can be used for adoptive transfer treatments, provide a sustained supply of cytotoxic NK cells without a donor and are maintained using good manufacturing practice (GMP) [14]. Moreover, genetic modification of NK cells receptors can also be used to optimize tumor targeting of NK cells [9].

Focused ultrasound (FUS) with microbubbles has been used to promote delivery and targeting of genes, pharmaceuticals, and adoptive cell transfer therapies [10]; and as a treatment in itself. Using low power FUS (0.6 & 1.4 MPa peak-rarefactional acoustic pressures) in a mouse tumor model, Liu *et al*. observed some tumor regression as well as transient increases in infiltration of non-T regulatory tumor infiltrating lymphocytes and longer term infiltration (>3 days) of CD8^+^ cytotoxic T-lymphocytes [15]. They suggest that this response is related to vascular permeability perturbations known to be associated with FUS [16–21] that could induce long term physiological changes. In studies of temporary blood-brain-barrier (BBB) opening with FUS, histology indicates little or no tissue structural perturbations at maximum absolute pressures less than 0.6 MPa with duty cycles approximately 5% that produce stable but not inertial microbubble cavitation [21–25]. Because local BBB permeability changes persist for several days, there are likely physiological/biochemical as well as physical changes induced by FUS [26]. Focused ultrasound has been shown to temporarily open the blood-brain-barrier (BBB) and promote successful penetration of targeted NK-92 cells expressing a chimeric HER2 antigen receptor (NK-92-scFv(FRP5)-zeta cells) and accumulation in HER2-expressing human breast metastasis model in rodents [27].

To be potent in treating solid tumors, NK cells must accumulate at the tumor site so that their cytotoxic effects will have maximum impact. In this study, we examined whether low dose FUS with microbubble stable cavitation (*ldb*FUS) can facilitate accumulation of human NK cells in a mouse xenograft of human colorectal adenocarcinoma (carcinoembryonic antigen (CEA)-expressing LS-174T) model in the presence of immunocytokine (ICK), hT84.66/M5A-IL-2 (M5A-IL-2). ICKs are a fusion of antibody-specific monoclonal antibody (mAb) and immune stimulating cytokine [28]. M5A-IL-2 is a fusion of a humanized anti-CEA mAb to IL-2. There are two roles of the ICK. First, the administration of ICK to mice is needed to stabilize NK cell viability and expansion *in vivo*. Miller and colleagues demonstrated that *ex vivo* activated NK cell expansion *in vivo* decreased by 90% one week after cytokine administration was discontinued [29]. Second, the ICK was used to target the tumor expressing CEA via the antibody portion M5A, and home in the targeting of NK cells, which express IL-2 receptors, to the tumor site via the cytokine IL-2. NK cell accumulation was assessed *in vivo* by first labeling NK cells with ferumoxytol (an FDA-approved ultra-small superparamagnetic iron oxide (USPIO) nanoparticle suspension) and then monitoring them using MRI.

## Materials and Methods

### Animal Model

The Institutional Animal Care and Use Committees (IACUC) of the California Institute of Technology and City of Hope approved this research study. All procedures were approved and conformed to the guidelines set out by the IACUC of both California Institute of Technology and City of Hope. NOD.Cg-Prkdc^scid^Il2rg^tm1Wjl^/SzJ (NSG) female mice (at least 10 weeks old from JAX breeding stock) were subcutaneously (s.c.) injected with LS-174T tumor cells (6x10^5^ cells in 0.2 ml) at both right and left lower flank sites. Optimal tumor sizes (~200–500 mm^3^) were achieved approximately 12 days post implantation. On the day of the *ldb*FUS and imaging, animals were given intravenous immunoglobulin (IVIG, 0.9 mg, i.p.) and immunocytokine (ICK, M5A-IL-2, 50μg, s.c.) 3–4 hours and 1 hour prior to intravenous tail vein injection of ferumoxytol-labeled NK cells (Fe-NK), respectively. Twenty-two animals received higher power *ldb*FUS/0.50MPa on one of the tumors during the combined microbubble/Fe-NK cell injection, while the contralateral tumor served as a control. Ten animals underwent the same procedure at a lower power *ldb*FUS/0.25MPa.

### Tumor Groups

All animals had bilateral LS-174T tumors on the high flank and received a dose of 10^7^ Fe-NK cells with the Optison microbubbles during *ldb*FUS. Tumors were grouped as follows: (+)*ldb*FUS/0.50MPa (n = 22), (-)*ldb*FUS/0.50MPa (n = 22), (+)*ldbFUS*/0.25MPa (n = 10), and (-)*ldb*FUS/0.25MPa (n = 10).

### Cells and Culture Conditions

#### Tumor Cells

Firefly luciferase expressing human colorectal adenocarcinoma cells (LS-174T) were collected in a suspension by trypsinizing culture after rinsing with DPBS (Dulbecco’s phosphate-buffered saline). The suspended cells were collected in culture media in a single 50ml conical tube and spun at 1200 rpm for 5 min in a bucket rotor centrifuge to remove trypsin from media. After centrifugation the supernatant was discarded and the cell pellet was re-suspended to the concentration of 3x10^6^/ml in 1% HSA in HBSS (human serum albumin/Hank’s buffered salt solution). Suspension in the conical tube was placed on ice.

#### Natural Killer Cells

Natural killer cells were purified from peripheral blood mononuclear cells (PBMC) that were isolated from whole blood by gradient centrifugation using Histopaque 1077 (Sigma-Aldrich). NK cells were purified from PMBC by negative selection using EasyStep Human NK Cell Enrichment Kit (Stem Cell) following the manufacturer’s instructions. After purification, NK cells were mixed with irradiated K562 cells transfected with murine IL-21 (obtained from Dean A. Lee, MD Andersen Cancer Center, Houston TX, in June 2012) at ratio of 1:2 (NK:K562) and co-cultured at 0.125x10^6^ NK/ml in NK expansion medium (RPMI-1640 + 5% human AB serum + 1x GlutaMax and 5ng/ml rhIL2). Cells were fed every 2–3 days and after 14 days, NK cells were frozen at 5x10^7^/vial in 90% heat inactivated Human AB serum and 10% DMSO. For each experiment NK cells were reactivated and expanded by co-culture with feeder cells and rhIL2 for a maximum of 2 weeks. The cells were shown to express CD56 by FACS analysis and were able to lyse LS-174T cells (84% lysis at 8:1 NK: target ratio). Isolated NK cells were expanded, purified and assessed following previously published standard protocol [11].

### NK Cell Labeling

#### Labeling with Ferumoxytol

NK cells were labeled with USPIO ferumoxytol (100 μg/ml, Feraheme, AMG Pharmaceuticals, hydrodynamic diameter 30 nm) by incubating them in medium containing clinical grade protamine sulfate (40 μg/ml, APP Pharmaceuticals) as a transfection agent for 2 hours [30]. After incubation, NK cells were washed with HBSS and centrifuged at 400 g for 5 min, twice. 10^7^ NK cells were re-suspended in 100μl volume of HBSS for use for each subject. NK cell viability was > 90% before and after labeling, with a labeling efficiency of >95%. Inductively Coupled Plasma Mass Spectrometry (ICP-MS) was performed and the average concentration of iron per NK cell was determined to be 0.1 (0.09–0.14) pg/cell. We and others observed no deleterious effects on NK cell viability at this low iron content [31, 32].

#### Labeling with CFSE

To obtain an independent measure of NK cell tumor accumulation, two NSG mice bearing bilateral flank LS-174T tumors underwent the same *ldb*FUS procedure but received NK cells that were labeled with carboxyfluorescein diacetate succinimidyl ester (CFSE, CellTrace^TM^, Life Technologies). CFSE was thawed for 1 hr prior to use with care to limit light exposure to any sample containing CFSE. NK cells were aliquoted from expanded stock, washed twice with HBSS and centrifuged at 400 g for 10 min, and resuspended to 20x10^6^ cell/ml in HBSS. After cell viability counts, NK cells were incubated in 5 μM CSFE for 15 min in the dark at 37°C. Following incubation, labeled NK cell were centrifuged at 400 g for 5 min, resuspended in 0.5 ml HBSS + 1% HSA, and incubated again at 37°C for 30 min to ensure complete modification of CFSE. The cell suspension was centrifuged at 400 g for 5 min and resuspended to 100x10^6^ cells/ml in 1% has in DPBS. 100 μl of the cell suspension was drawn for the final injection dose.

### Low Dose Bubble-mediated Focused Ultrasound (*ldb*FUS)

We used a 6.4 cm 510 kHz transducer, integrated hydrophone (H107 & Y107 Sonic Concepts, Bothell, WA) and a coupling cone filled with degassed water. Agilent 33220A function generator drove a Henry Electronics 50 watt amp and a Tektronics TDS3014B oscilloscope in FFT mode monitored the hydrophone. An RP Acoustics (Leutenbach, DE) PVDF hydrophone (RP24I) was used to calibrate the negative peak rarefication pressure of the H107 transducer. In a tank of degassed water, we placed the RP24I tip 5 mm from the coupling membrane of the coupling cone (filled with degassed water) housing the H107 transducer. 5 mm was chosen as that is the approximate expected tumor radius. By varying the mV_rms_ from the function generator driving the power amplifier and assuming and 18% attenuation, we found that 40 mV_rms_ and 150 mV_rms_ will deliver 0.25 MPa and 0.5 MPa respectively to the mouse tumor.

**Fig 1:**
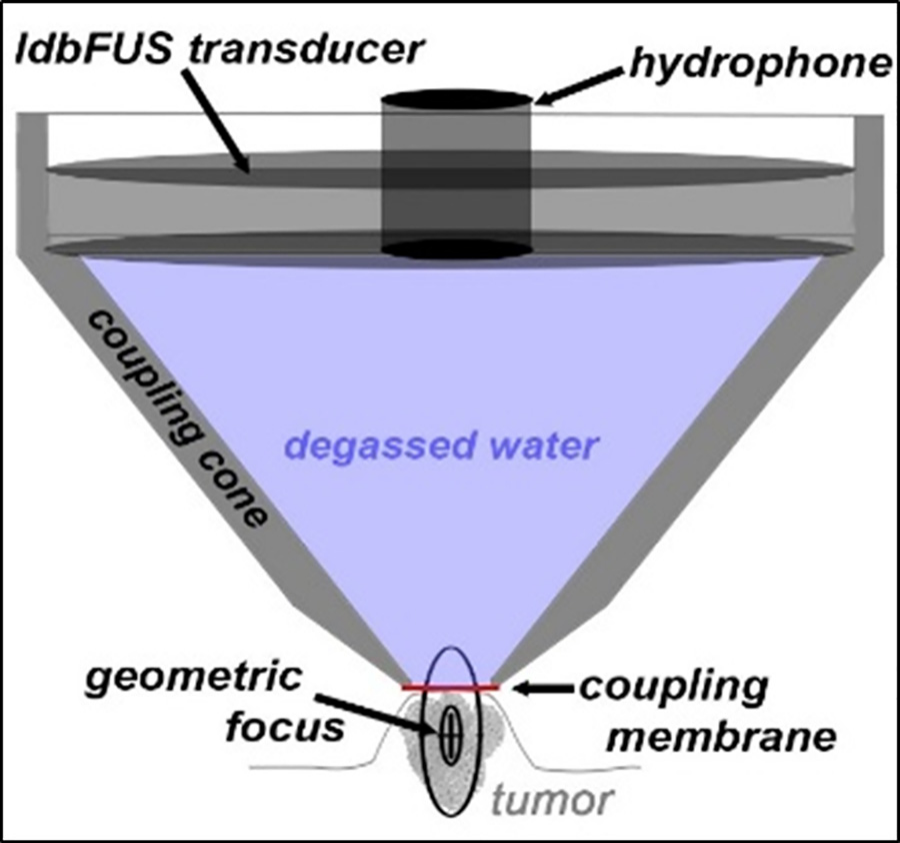
Diagram of *ldb*FUS setup. *ldb*FUS transducer and hydrophone (Sonic Concepts H107 & Y107) are fixed in a plastic coupling cone filled with degassed water and sealed with a thin membrane. Ultrasound gel between membrane & tumor ensure good sonic contact. Focal region of *ldb*FUS transducer is ~5mm diameter & 15mm long; centered below the coupling membrane and within the tumor.

While under anesthesia (1.5% isoflurane), the mouse was placed in the stereotaxic frame and the coupling cone was positioned on the tumor with ultrasound transmission gel (Aquasonic 100, Parker) (Fig 1). The transducer was driven at its center frequency (510 kHz) for 10 ms every second for 1 minute. During the 1 minute of ultrasound, 100 μl Optison (GE Healthcare) microbubbles plus 100 μl of ~10^7^ ferumoxytol-labeled NK cells (Fe-NK) or CFSE labeled NK cells (total volume of 200 μl) was delivered via tail vein catheter.

### MRI

2D Multi Gradient Echo (MGE) was performed on a 7T Bruker-Biospin scanner with the following parameters: repetition time (TR), 1500 ms, 6 echoes starting at 3.74 ms and spacing of 4.84 ms; field of view (FOV), 3.5 cm x 2.5 cm; spatial resolution, 0.150 mm x 0.150 mm; slice thickness, 0.75 mm; matrix size = 233 x 167; and 4 averages. The RF coil used for imaging was a 35 mm diameter quadrature volume coil (M2M Imaging Corporation, Cleveland, OH). Animals were induced and maintained with 1.5% isoflurane anesthesia and were placed in an acrylic cradle where body temperature was maintained using warm air and respiration was monitored (Biopac Systems). Acquisition was respiration-gated. Tumor size dictated the number of slices acquired to ensure full coverage of the tumor mass.

**Fig 2:**
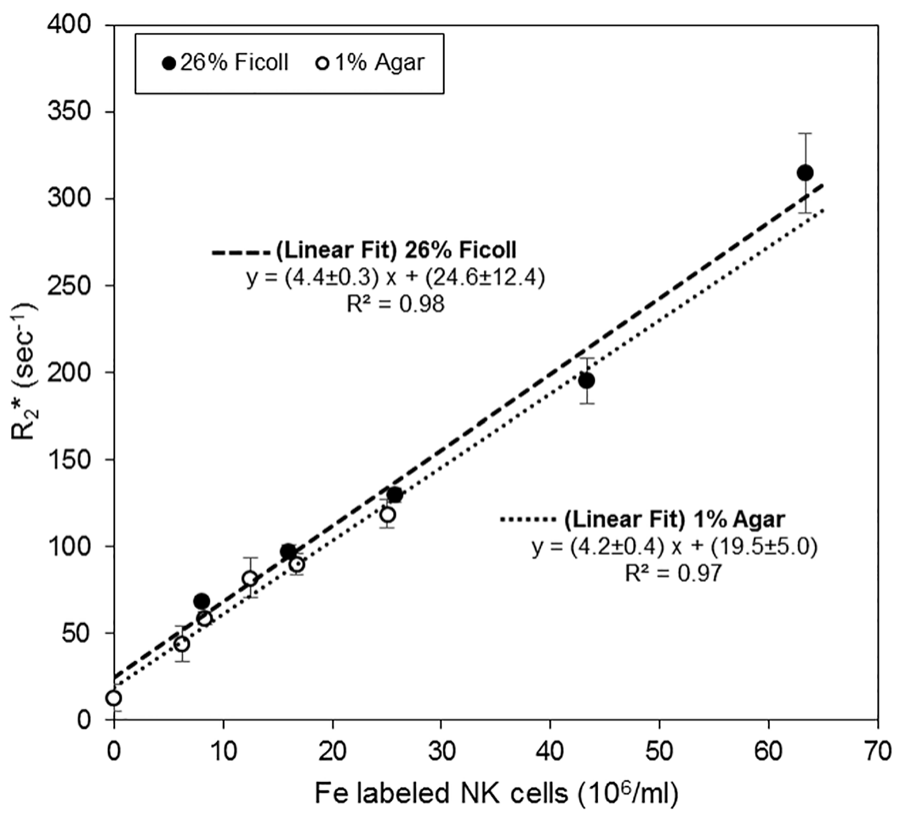
Relaxation rate is linear in Fe labeled NK cell concentration. Ferumoxytol labeled NK cells were suspended in both 26% Ficoll and 1% agar solutions and R_2_* determined at 7T using 2D MGE protocol (mean±SD). There is no significant difference between fitted slopes and intercepts of Fe-NK suspensions in 26% Ficoll and 1% agar solutions.

To determine *in vivo* concentrations of ferumoxytol labeled NK cells, we measured R_2_* relaxation rates as a function of NK cell concentration (Fig 2). R_2_* is linear with iron concentration in the ranges of interest and therefore with NK cell concentration. Ferumoxytol labeled NK cells were suspended in 26% Ficoll or 1% agar solution and R_2_* was determined using 2D MGE. For Fe-NK suspended in 1% agar the following parameters were used: repetition time (TR) = 1500 ms, 6 echoes starting at 3.74 ms and spacing of 4.84 ms; field of view (FOV) = 3.5 cm x 2.5 cm; spatial resolution = 0.150 mm x 0.150 mm; slice thickness = 0.75 mm; matrix size = 233 x 167; and averages = 4. For Fe-NK suspended in 26% Ficoll the following parameters were used: TR = 1500 ms, 16 echoes starting at 4.27 ms and spacing of 5.88 ms; FOV = 6 cm x 2 cm; spatial resolution = 0.200 mm x 0.200 mm; slice thickness = 3 mm; matrix size = 300 x 100; and averages = 4. The RF coil used for imaging was a 35 mm diameter quadrature volume coil (M2M Imaging Corporation, Cleveland, OH).

To determine whether the *ldb*FUS/Fe-NK (0.50 MPa) induces changes in the tumor environment, a subset of the NSG mice underwent diffusion weighted imaging to determine changes in apparent diffusion coefficient (ADC) before and after *ldb*FUS/Fe-NK administration. ADC has been correlated with tumor cellularity, extracellular volume, edema and tumor grade, and even to tumor treatment responses [33–35]. Diffusion MRI was acquired with the following parameters: TR/TE = 2250 ms / 27.5 ms, with six *b* values = 0, 100, 200, 400, 600, and 800 s/mm^2^ acquired in 3 orthogonal directions; FOV = 3.2 cm x 2 cm; slice thickness = 1.5; spatial resolution = 150 mm x 299 mm; matrix size = 233 x 67; NA = 1.

#### MRI Analysis

Using ROCKETSHIP v.1.1 code [36] in MATLAB (R2014b), T_2_* and ADC maps were generated through a pixel-by-pixel exponential fitting of signal intensities across the different TE times and *b* values, respectively. In each tumor at each time point a region of interest (ROI) was manually drawn using ImageJ2 [37] over every tumor slice so that the ROI encompassed the whole tumor. R_2_* maps were generated by taking the inverse of the T_2_* map (R_2_* = 1/T_2_*). Histogram frequency distribution of R_2_* values (range = 0–200 sec^-1^, bin width = 1) were obtained for each tumor at each time point. The R_2_* geometric means were obtained by fitting the R_2_* histogram to a lognormal distribution (MATLAB). Because tumors have a nonzero ‘background’ R_2_*, the change in R_2_* (ΔR_2_*) measures the concentration of NK cells that accumulates in the tumor ROI. ΔR_2_* is calculated by taking the difference between the R_2_* values at the time points post Fe-NK injection and the pre-injection R_2_* values.

### Histological Analysis

Following the 6 hr time point post *ldb*FUS/Fe-NK administration, 3 NSG mice that received 0.50 MPa *ldb*FUS were euthanized by CO_2_ inhalation and underwent cardiac perfusion using 4% paraformaldehyde in 0.1 M phosphate buffer saline. The (+)*ldb*FUS and (-)*ldb*FUS tumors and spleen were removed and placed in PBS with 0.01% sodium azide and stored at 4°C. Tissues were prepared in an OCT^®^ (frozen tissue matrix) block and were stored at -80°C. Prior to cutting sections, the block was allowed to equilibrate to -20°C. Tissue blocks were sectioned at 18μm thickness. Sections were stained with horseradish peroxidase (HRP) with anti-CD56 (NK cell marker) and Prussian blue for the detection of iron oxide ferumoxytol particles. Sections from the same blocks were also stained for anti-CD56 fluorescence markers (546 nm) and DAPI. Images from sections were captured using Zeiss upright widefield microscope and ZEN software. Fluorescent positive-anti-CD56 counts were quantified using ImageJ2 Analyze Particles from (+)*ldb*FUS and (-)*ldb*FUS tumor sections. The 2 mice that received NK-CSFE cells underwent the same procedure for tissue preparation and sectioning. NK-CSFE images were acquired using Hamamatsu NanoZoomer 2.0 HT and positive-NK-CSFE counts were quantified using Image Pro Premier 9.1 software.

### Statistical Analysis

MATLAB (R2014b) was used for all statistical analyses. ΔR_2_* values of the different groups at each time point were represented in box plots. Group comparisons were performed using Kruskal-Wallis H test with a Bonferroni post hoc test. Planned group comparisons were performed using the Wilcoxon rank sum test. Comparison between pre and post time points used the Wilcoxon signed-rank paired test. Mean ± standard deviation (SD) were presented for NK cell concentration accumulation, determined from ΔR_2_*or quantitation from anti-CD56 and CSFE histology. Student’s t-tests were used to analyze NK cell accumulation in tumors following *ldb*FUS/Fe-NK treatment. Statistical significance was noted if p < 0.05.

## Results

We first established that R_2_* is a linear function of ferumoxytol labeled NK cell concentration over the concentration range of interest in the study (Fig 2). Various concentrations of ferumoxytol labeled NK cells were suspended in either 26% Ficoll or 1% agar solution and R_2_* values were determined at 7T. For every increase of 10^6^ NK cells per ml we observed an increase in R_2_* of 4.4±0.3 sec^-1^ in 26% Ficoll and 4.2s±0.4 sec^-1^ in 1% agar solutions. There are no significant differences between fitted slopes and intercepts of Fe-NK suspensions in 26% Ficoll and 1% agar solutions.

**Fig 3:**
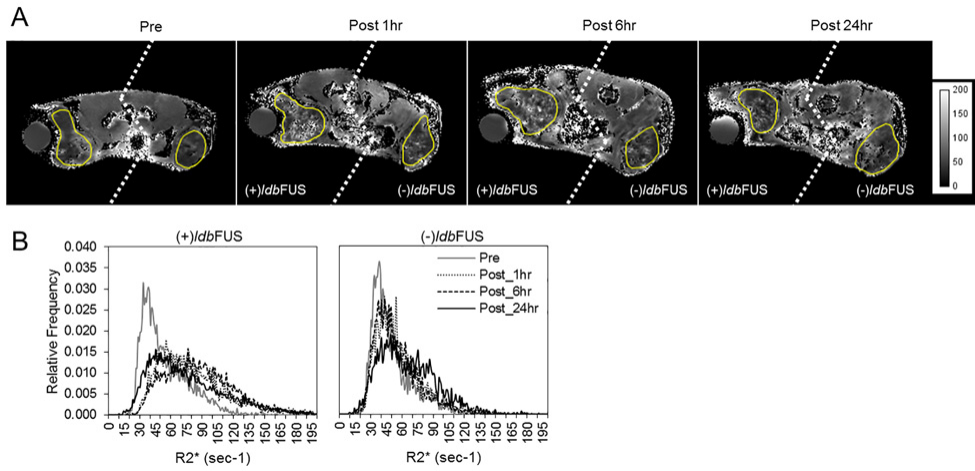
R_2_* maps and tumor histograms monitor changes in NK cell concentration. (A) Representative parametric R_2_* images (axial slices) of an NSG mouse bearing bilateral flank LS-174T tumors before (pre), 1hr, 6hr, and 24hr post *ldb*FUS/Fe-NK administration. *ldb*FUS was induced at peak rarefication pressure of 0.50MPa. Yellow outlines show tumor regions of interest. Note that at some imaging time points left and right tumors were not in the same field of view–thus left & right sides (dashed line) may be from slightly different axial slices. Units in sec^-1^. (B) Histogram showing relative frequency R_2_* distributions of whole tumors from (A). The histogram shifts to increasing values of R_2_* with time in (+)*ldb*FUS tumor.

**Fig 4:**
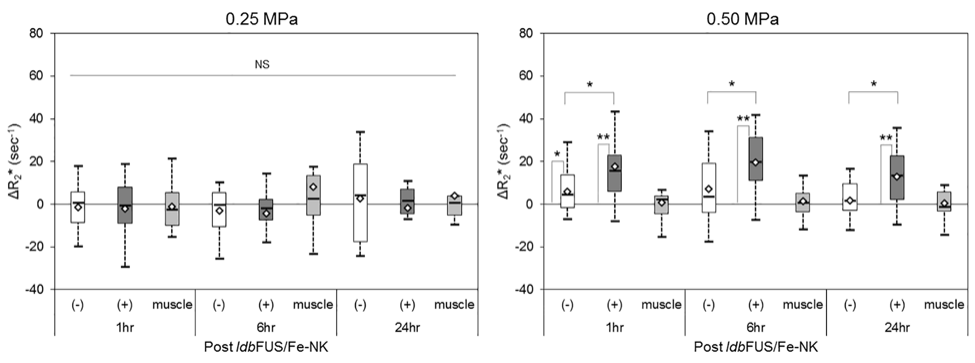
Boxplots of ΔR_2_* for LS-174T tumors without (-) and treated with (+) *ldb*FUS and non-tumoral back muscle regions in NSG mice that received either low power (0.25MPa, left) or higher power (0.50MPa, right) *ldb*FUS. Thick center lines show the medians; box limits indicate the 25th and 75th percentiles as determined by R software; whiskers extend to 1.5 times the interquartile range; diamond-shaped markers represent sample means. A Kruskal Wallis H test showed that there was a statistically significant difference in ΔR_2_* between the four groups (p<0.00001). Post hoc comparisons (pre vs. post or (-) vs. (+) *ldb*FUS) that showed significant differences are indicated by *p<0.05 and **p<0.01. NS = no significant difference.

We performed *ldb*FUS at two pressure levels: 0.25 MPa and 0.50 MPa. In all cases, only one of the bilateral tumors received sonication; the contralateral tumor serving as an internal control. All animals tolerated the ldbFUS procedure, the cell injection, and subsequent longitudinal MR imaging without complications. Representative pre and post *ldb*FUS/Fe-NK R_2_* maps from an NSG mouse that received the higher pressure *ldb*FUS (0.50 MPa) are shown in Fig 3A. Post treatment distributions in the (+)*ldb*FUS tumor region showed increasing values of R_2_*, as shown in Fig 3B. Fig 4 shows box plots of the ΔR_2_* geometric means of each tumor group and control non-tumoral back muscle region. A Kruskal Wallis H test showed that there was a statistically significant difference in ΔR_2_* between the four groups (p<0.00001). Post hoc testing showed that ΔR_2_* values of (+)*ldb*FUS/0.50MPa tumors were significantly higher than the contralateral (-)*ldb*FUS/0.50MPa tumors at 1 hr (p = 0.014), 6 hr (p = 0.012), and 24 hr (p = 0.013). (+)*ldb*FUS/0.50MPa tumor ΔR_2_* values were also significantly higher than the (-)*ldb*FUS/0.25MPa at 1 hr (p<0.01), 6 hr (p<0.01) and 24 hr (p = 0.03). (+)*ldb*FUS/0.50MPa tumors were significantly different from (+)*ldb*FUS/0.25MPa in the early 1 hr and 6 hr post times (p<0.01). Use of the higher pressure *ldb*FUS/0.50MPa protocol induced significant increases in ΔR_2_* that persisted up to 24 hr post treatment. The control contralateral tumors that did not receive the higher pressure *ldb*FUS/0.50MPa procedure also showed significant increase in ΔR_2_* at the 1hr time point compared to the baseline values (p<0.05). No significant differences from pre values were observed in either 0.25 MPa tumor group across time or between groups. Control non-tumoral back muscle ΔR_2_* showed no significant changes between pre and post *ldb*FUS/Fe-NK time points, indicating the ΔR_2_* was tumor-specific.

**Fig 5:**
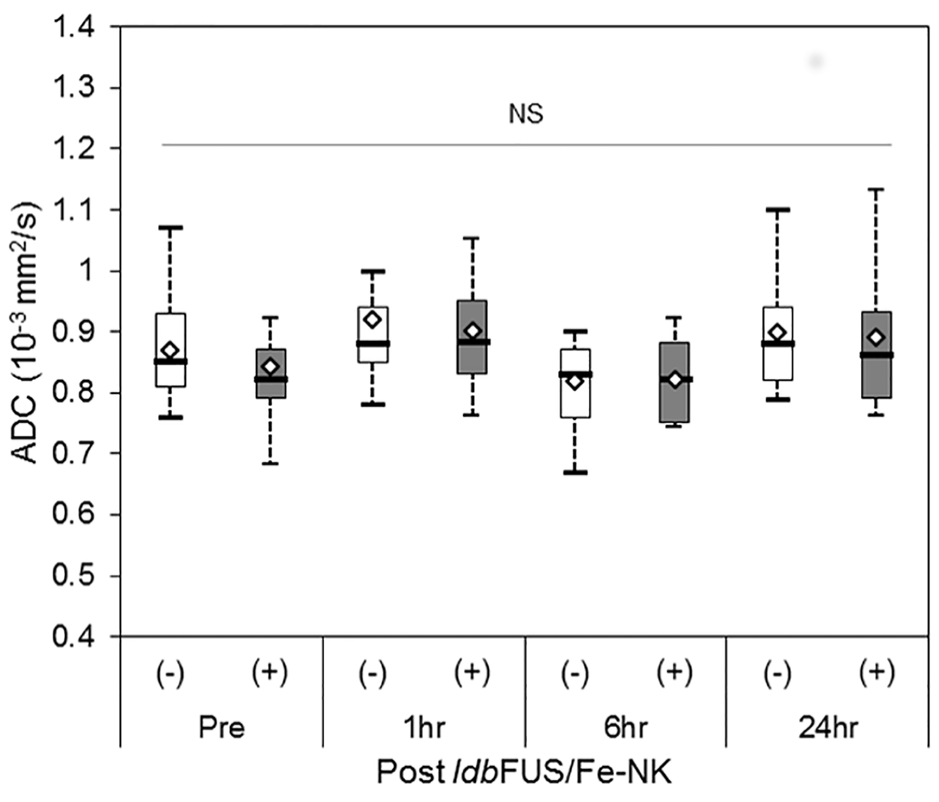
Boxplots of Apparent Diffusion Coefficient (ADC) values for a subset of LS-174T tumors (n = 12) without (-) and treated with (+) *ldb*FUS/0.50MPa in NSG mice. Thick center lines show the medians; box limits indicate the 25th and 75th percentiles as determined by R software; whiskers extend to 1.5 times the interquartile range; diamond-shaped markers represent sample means. There were no significant changes in ADC values between *ldb*FUS treated and non treated tumors and across time points.

Additionally, a subset of the NSG animals that received *ldb*FUS/0.50MPa underwent diffusion weighted imaging to determine changes in apparent diffusion coefficient (ADC), reflective of increased water content and edema. ADC results showed no significant differences between (-)*ldb*FUS and (+)*ldb*FUS tumors across all time points (Fig 5).

**Fig 6:**
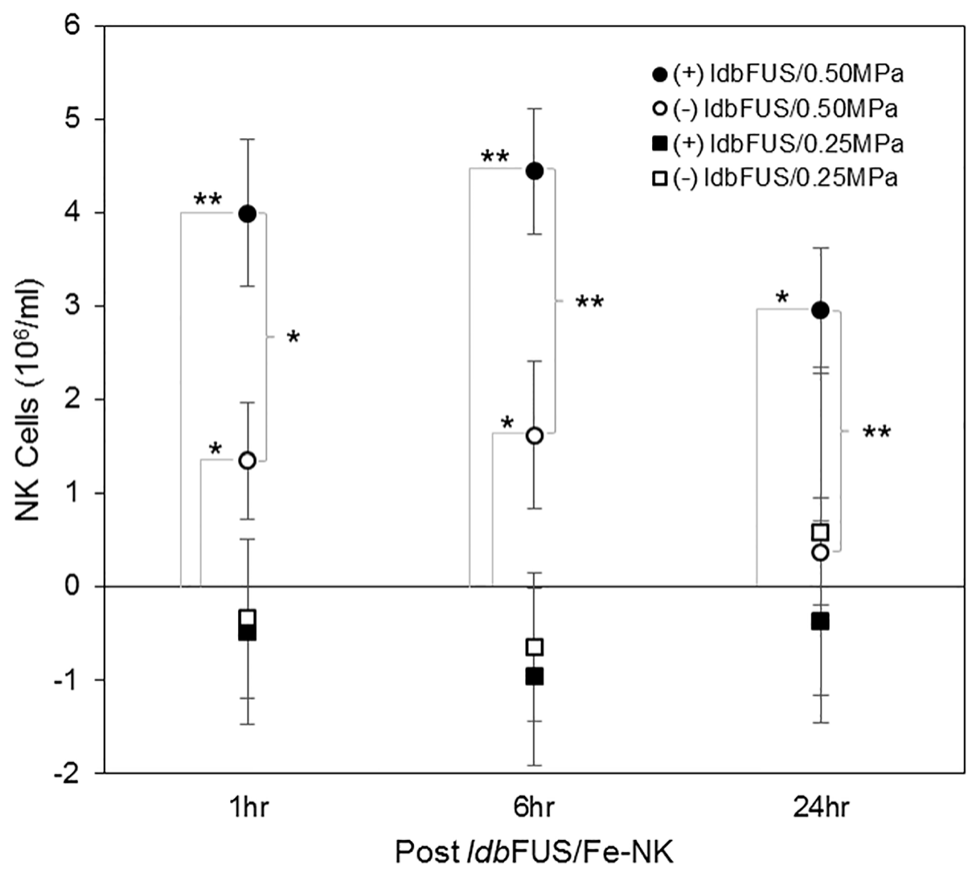
NK cell concentration in tumors determined from ΔR_2_*. Although NK cell tumor distributions are heterogeneous as seen in Fig 3, R_2_* geometric means were obtained by fitting the R_2_* histogram to a lognormal distribution. NK cell concentrations (10^6^/ml, mean±SD) in tumor tissue obtained from ΔR_2_* determination and the linear relationship between R_2_* and NK cell concentration shown in Fig 2. Planned comparisons were performed using the Student’s t-test. Significant differences are indicated by *p<0.05 and **p<0.01.

Fig 6 illustrates the NK cell concentration (10^6^/ml, mean±SD) in tumors determined from the MRI signal ΔR_2_* (using information from the 26% Ficoll R_2_* vs NK cell concentration curve in Fig 2). Significantly more NK cells per volume of tumor tissue accumulated at the 1 hr, 6 hr, and 24 hr time points in (+)*ldb*FUS/0.50MPa tumors (1 hr: 4.18±0.17, 6 hr: 4.65±0.15, 24 hr: 2.78±0.15 10^3^/mm^3^) than in the control contralateral (-)*ldb*FUS/0.50MPa tumors (1 hr: 1.41±0.14, 6 hr: 1.70±0.18, 24 hr: 0.33±0.11 10^3^/mm^3^) (p<0.01, (+)*ldb*FUS vs (-)*ldb*FUS). Overtime, the increased number of NK cells that accumulated in the (+)*ldb*FUS/0.50MPa tumors was statistically significant (p<0.01). The control (-)*ldbFUS*/0.50MPa tumors also showed NK cell presence only 1 hr and 6 hr after the administration of *ldb*FUS/Fe-NK. No significant accumulation of NK cells were observed in either (+)*ldbFUS*/0.25MPa or (-)*ldbFUS*/0.25MPa tumors.

**Fig 7:**
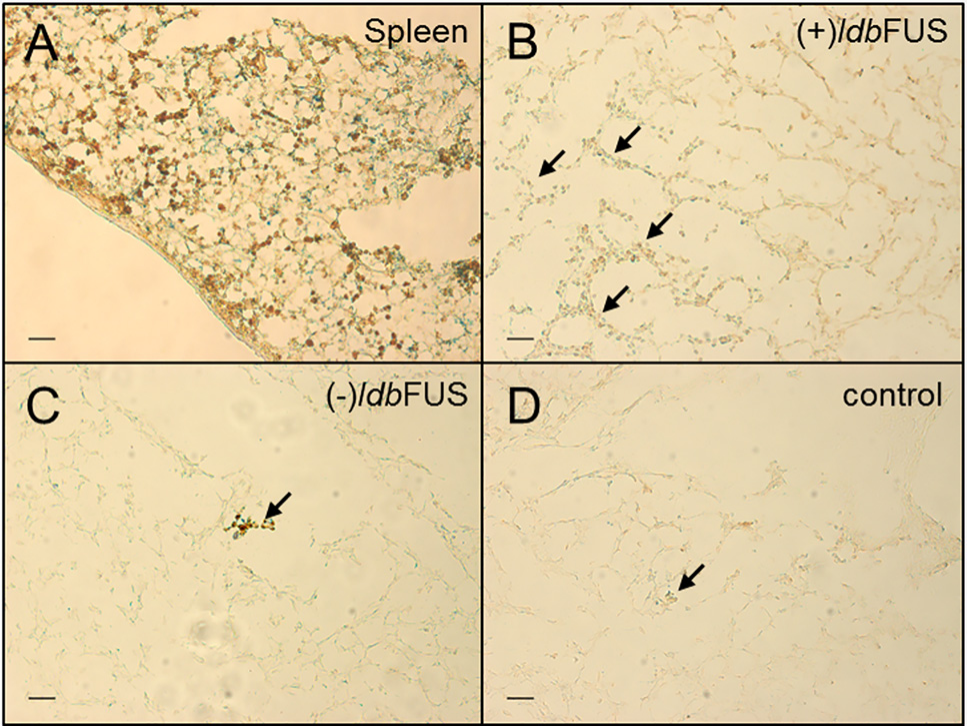
Anti-CD56-HRP plus Prussian Blue staining of spleen and tumor regions show accumulation of NK cells. Spleen (A) and tumor regions (B, C) are from an NSG mouse. (B) Tumor region receiving *ldb*FUS (0.5MPa) and (C) tumor contralateral to B. (D) Tumor region from an NSG that received no *ldb*FUS treatment. Scale bar = 50 μm. Arrows show greater number of colocalization of positive CD56 (brown) and positive iron (blue) staining from *ldb*FUS administered tumor than tumors without treatment.

**Fig 8:**
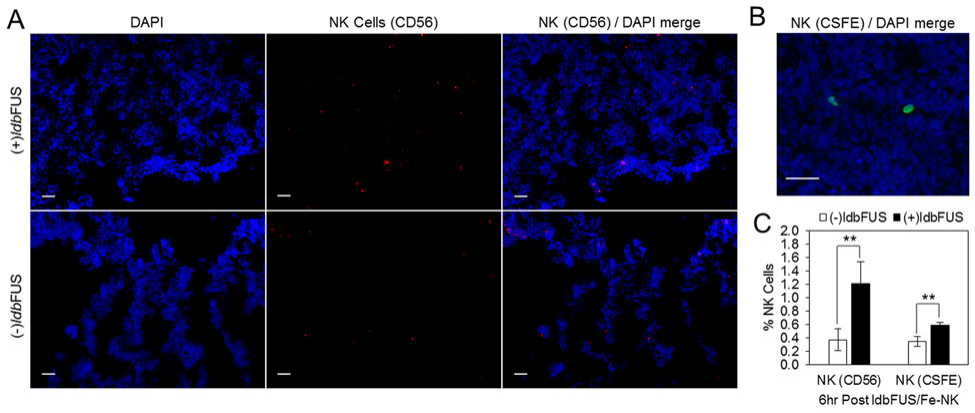
Quantitation of % NK cells from fluorescent staining of tumors from an NSG mouse. (A) Fluorescent NK cells (CD56, red) and DAPI (blue) staining of tumors from an NSG mouse: upper panel from tumor administered *ldb*FUS/0.50 MPa; lower panel from contralateral tumor that was not treated. Scale bar = 50 μm. (B) A sample section with positive-NK-CSFE (green) in tumor tissue (DAPI, blue). Scale bar = 50 μm. (C) Mean±SD Percent % NK cells determined from CD56 and CSFE fluorescent staining. % NK cells = number of NK cells / total number of cells (determined from DAPI stain). Planned comparisons using t-test showed a significant difference between (-)*ldb*FUS and (+)*ldb*FUS, **p<0.01.

Following animal euthanasia, histological tumor sections with anti-CD56-HRP and Prussian blue staining showed ferumoxytol labeled NK cells in the spleen and tumor regions (Fig 7). Fig 8A shows representative tumor sections stained with fluorescent anti-CD56 (red) and DAPI (blue) that received *ldb*FUS/0.50MPa on one tumor (upper panel) and sections from the contralateral control tumor (lower panel) from an NSG mouse. Quantitation of percent NK cells (mean±SD, CD56 positive counts per total tumor cell count) showed that (+)*ldb*FUS/0.50MPa (1.21±0.32%) had significantly higher counts than the (-)*ldb*FUS/0.50MPa control tumor (0.37±0.17%) (Fig 8C).

We performed another independent measure of NK cell accumulation in tumors after *ldb*FUS induction. NK cells labeled with CSFE were simultaneously injected with microbubbles during higher power *ldb*FUS/0.50MPa. A sample section with positive-NK-CSFE staining in tumor tissue is shown in Fig 8B. Quantitation of percent NK cells (mean±SD, NK-CSFE positive counts per total tumor cell count) revealed (+)*ldb*FUS (0.59±0.05%) had a greater number of NK presence than (-)*ldb*FUS (0.35±0.07%), (Fig 8C, p<0.01).

## Discussion

Tumor accumulation and infiltration of NK cells are crucial for effective NK cell based therapy. Our study shows that administration of *ldb*FUS onto the tumor mass facilitates accumulation of adoptively transferred NK cells in a human cancer xenograft. LS-174T tumors treated with *ldb*FUS at 0.50MPa had substantial accumulation of NK cells compared to non *ldb*FUS treated tumors, as measured by *in vivo* MRI ΔR_2_* increases following administration of ferumoxytol-labeled NK cells. Quantitation of CD56 NK cell marker and of fluorescently labeled NK cells confirmed a consistent 2-fold increase in NK cell accumulation in tumors treated with *ldb*FUS (0.5MPa). In contrast, ferumoxytol-labeled NK cells showed no specific accumulation when tumors were treated with lower power *ldb*FUS (0.25MPa).

Focused ultrasound (FUS) uses acoustic wave propagation to deposit energy in tissue for therapeutic purposes instead of acquiring images. High intensity focused ultrasound (HIFU) is mainly used clinically for thermal ablation of targeted tissue [38, 39], which can result in altering the local immune environment for pro-therapeutic effects. Lower intensity FUS has also been demonstrated to promote drug delivery when paired with microbubbles during FUS (*ldb*FUS), induce immunological responses, and promote homing of various cells (CD8+ cytotoxic T-lymphocytes, dendritic cells, NK cells, neutrophils, and macrophages) [40–44]. Furthermore, microbubbles undergoing stable cavitation can produce fluid shear stresses that can induce pro-extravasation of activated leukocytes *in vitro* (increased adhesion, deformability, motility, and transmigration) [10]. Interestingly, we observed increased ΔR_2_* signal in the non-*ldb*FUS tumor from animals that received *ldb*FUS on the ipsilateral tumor at higher power, compared to animals that received the lower power ultrasound. Thus, with sufficient power, *ldb*FUS can have both a local and systemic effect that is tumor-specific. Power and length of application of *ldb*FUS, as well as relative timing of introduction of NK cells, are all variables that influence the relative (as well as absolute) importance of the local and systemic effects. We note that Alkins *et al*. showed that injection of NK-92 cells immediately prior to BBB disruption resulted in optimal translocation of HER2 specific NK-92 cells to HER2 expressing breast metastasis [10].

The use of ferumoxytol, a US FDA-approved iron supplement for iron deficiency in patients with chronic kidney disease, as a cell labeling agent supports ready clinical translation of noninvasive measurements of early tumor response to treatment using MRI. As a proof of concept, *in vivo* cell tracking using ferumoxytol has been demonstrated using adipose-derived stem cells implantation in osterchondral defects of arthritic joints with MRI [45]. The quantitative approach of generating relaxation rate maps to measure concentrations of cells has also been demonstrated by Sheu *et al*., who tracked super paramagnetic iron oxide (SPIO) labeled NK cells in hepatocellular carcinoma rats [46].

NK cells used in this study were of human origin and activated using rhIL-2 during expansion *in vitro* prior to adoptive transfer. Single doses of IVIG and ICK (M5A-IL-2) were administered prior to the adoptive transfer of NK cells in order to promote additional homing and activation of NK cells. Since NSG mice lack circulating IgG, IVIG was used to block neonatal Fc receptor (FcRn) clearance of the administered ICK via Fc:FcRn interaction and improve the circulating half-life of the ICK [47]. The M5A antibody portion of the ICK binds to the tumor associated carcinoembryonic (CEA) antigens of the LS-174T cells [48], which in turn are recognized by NK cell CD16 (FcγIIIR) receptors [49]. The IL-2 cytokine portion may further provide a costimulatory adjuvant signal for enhancing NK cell mediated antitumor response [28]. The level and frequency of this IVIG/ICK regimen that could result in optimal tumor trafficking of NK cells is under further investigation.

There are several methods to potentiate the killing activity of NK cells. KIR mismatch between allogeneic NK cells and host tumor cells can enhance tumor-killing ability of NK cells through the “missing-self” recognition process [12, 13]. Genetic engineering of NK cells either allogeneicly isolated or from cytotoxic NK cell lines adds a layer of optimization for enhanced tumor homing and killing [9]. Activation of NK cells is influenced by the balance of the inhibitory and activating receptors present on the surface of the cells. Activation can be potentiated through down-regulating the inhibitory receptors or overexpressing the activating NK cell receptors using small interfering RNA-based technologies [50, 51]. Genetic transfer of chimeric tumor-antigen-specific receptors on NK cells also enhances targeting of these cells [10, 31, 52]. Combining these techniques for enhancing NK cell activity with optimized immunocytokine regimen and *ldb*FUS induction would be a step towards successful multimodal immunotherapy.

We have demonstrated that NK cells homing to tumor regions can be potentiated by direct induction of *ldb*FUS onto the tumor mass and can be evaluated non-invasively using MRI. NK cells are already used clinically, MRI guided focused ultrasound [53] is approved for treatment of uterine fibroids and is in trials for treatment of several cancers, and several microbubble agents are approved for ultrasound imaging applications; thus all methods used in this study are currently in use clinically in some form, demonstrating the practical feasibility of translation into the clinic.

## Supporting Information

S1 Dataset Apparent Diffusion Coefficient Data. (XLSX)

S2 Dataset ΔR_2_* Data for *ldb*FUS/0.25MPa Group. (XLSX)

S3 Dataset ΔR_2_* Data for *ldb*FUS/0.50MPa Group. (XLSX)

S4 Dataset Data for Histological Quantification of % NK Cell Counts. (XLSX)

S5 Dataset R_2_* versus Fe-NK Cell Concentration Data. (XLSX)

S1 Fig Sample Positive-Anti-CD-56 Staining. (TIF)

S2 Fig Sample DAPI Staining. (TIF)

S3 Fig Sample Merged Image of Positive-Anti-CD-56 and DAPI Staining. (TIF)
