# Supplementary figures and images for: Low Dose Focused Ultrasound Induces Enhanced Tumor Accumulation of Natural Killer Cells
Source: PLoS One. 2015 Nov 10;10(11):e0142767. doi: 10.1371/journal.pone.0142767 (PMC4640510; doi:10.1371/journal.pone.0142767)

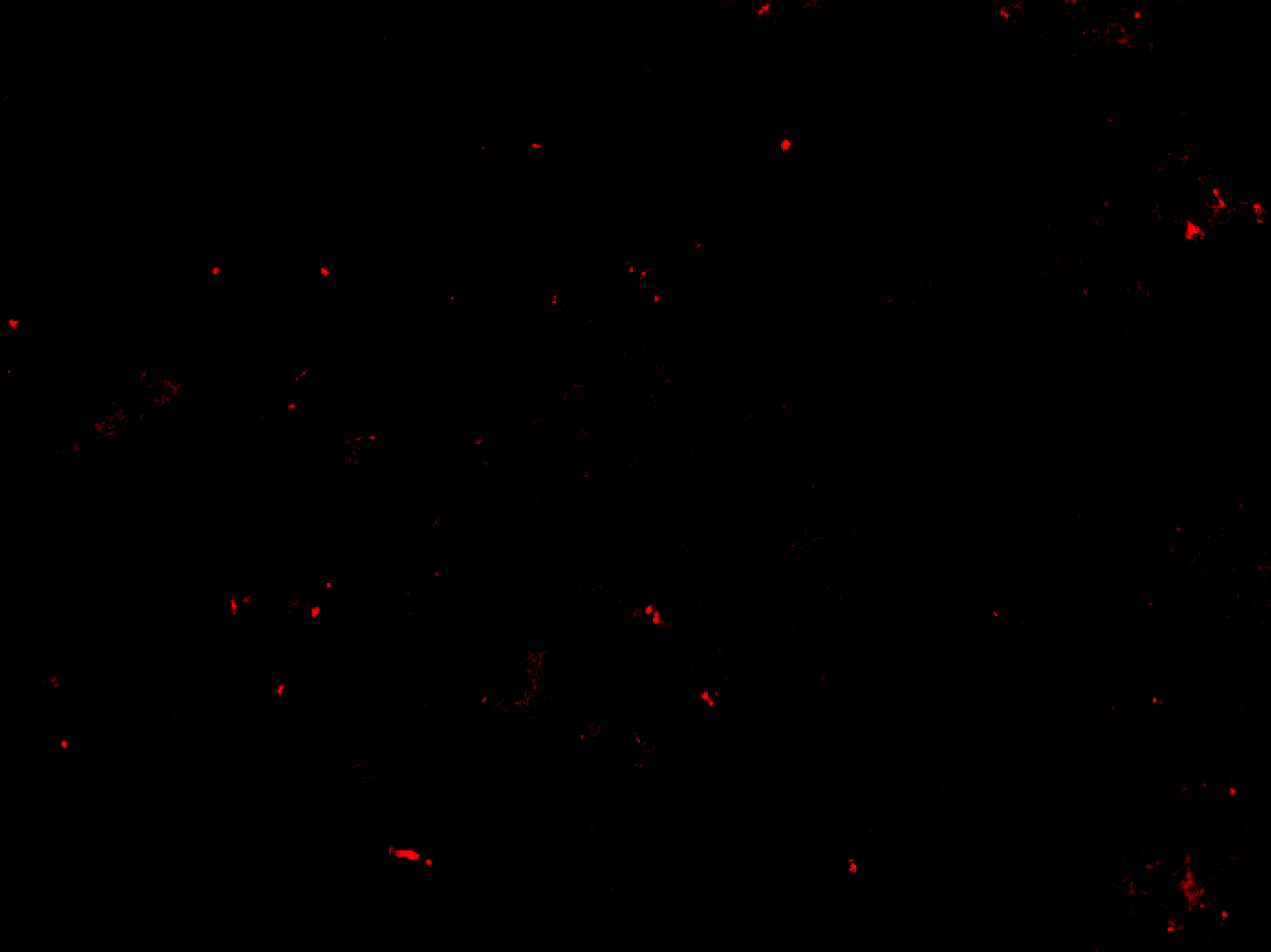

Supplement: S1 Fig — (TIF) [file pone.0142767.s006.tif]

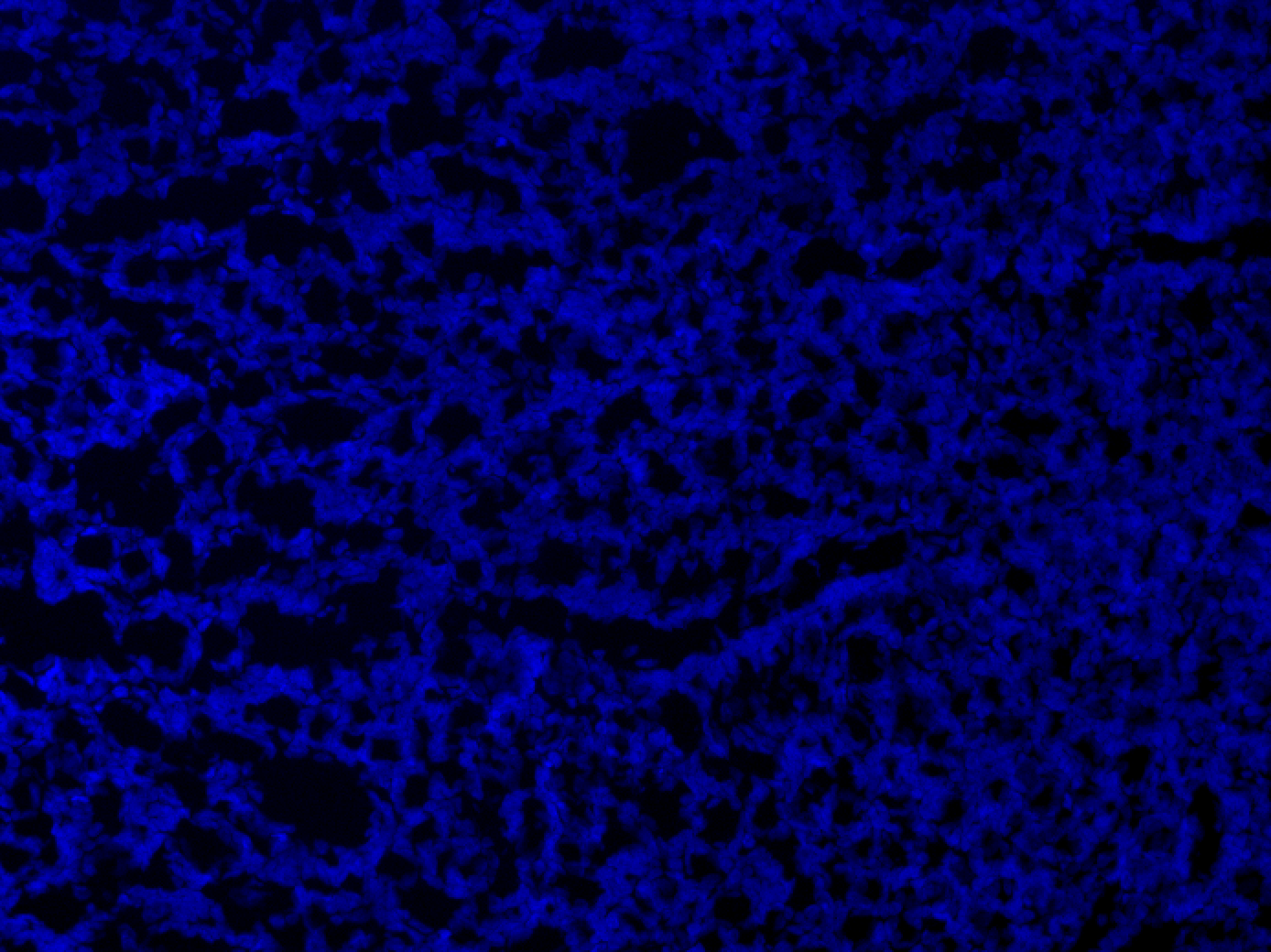

Supplement: S2 Fig — (TIF) [file pone.0142767.s007.tif]

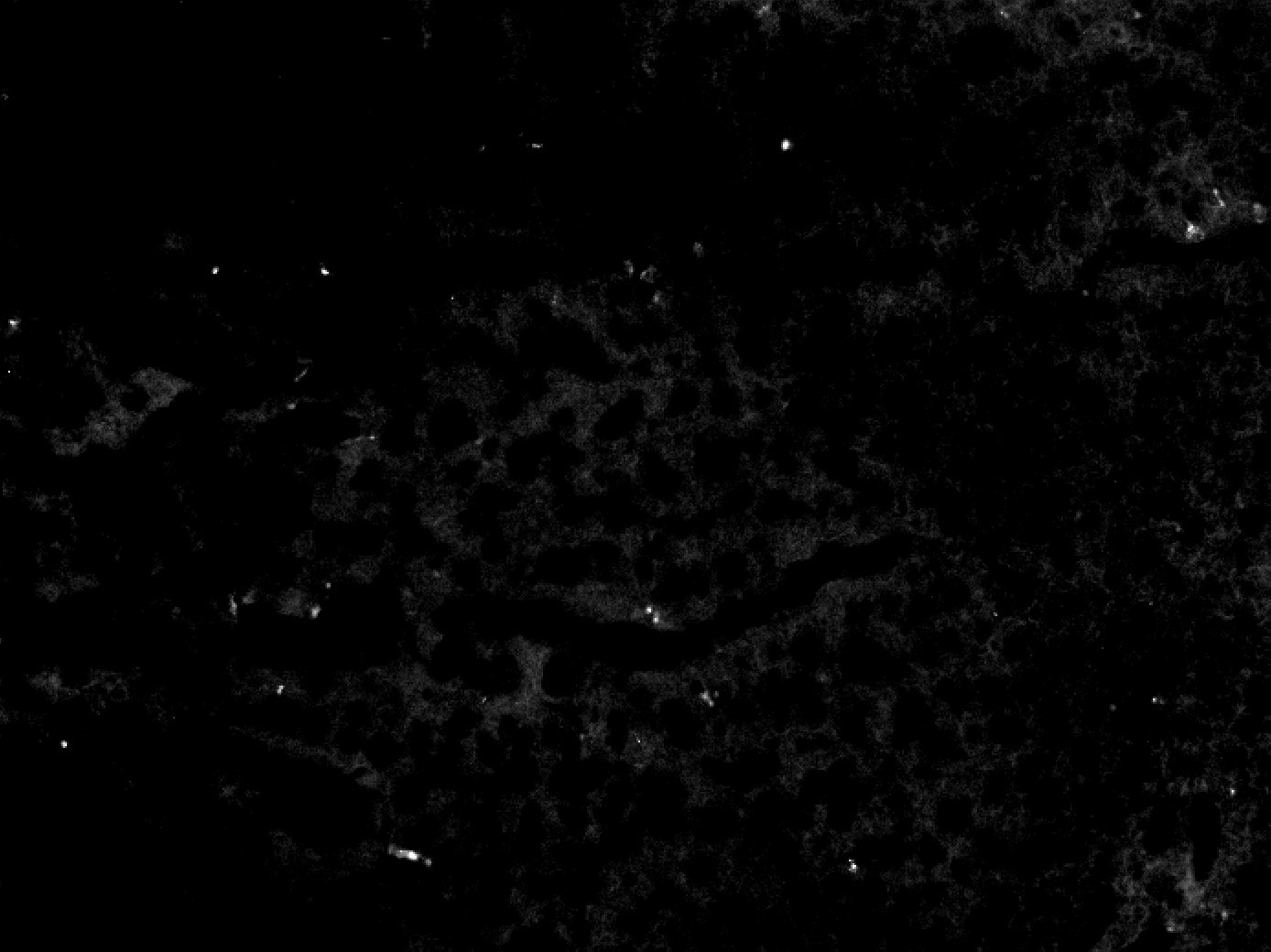

Supplement: S3 Fig — (TIF) [file pone.0142767.s008.tif]
